# Minimum Entropy Generation Rate and Maximum Yield Optimization of Sulfuric Acid Decomposition Process Using NSGA-II

**DOI:** 10.3390/e22101065

**Published:** 2020-09-23

**Authors:** Ming Sun, Shaojun Xia, Lingen Chen, Chao Wang, Chenqi Tang

**Affiliations:** 1 College of Power Engineering, Naval University of Engineering, Wuhan 430033, China; 17862989281@163.com (M.S.); 15994280441@139.com (S.X.); victoria329@163.com (C.W.); tangchenqi7@163.com (C.T.); 2 Institute of Thermal Science and Power Engineering, Wuhan Institute of Technology, Wuhan 430205, China; 3 School of Mechanical & Electrical Engineering, Wuhan Institute of Technology, Wuhan 430205, China

**Keywords:** finite-time thermodynamics, sulfuric acid decomposition, tubular plug-flow reactor, entropy generation rate, SO_2_ yield, multi-objective optimization

## Abstract

Based on the theory of finite-time thermodynamics (FTT), the effects of three design parameters, that is, inlet temperature, inlet pressure, and inlet total mole flow rate, of a tubular plug-flow sulfuric acid decomposition reactor on the total entropy generation rate (EGR) and SO_2_ yield are analyzed firstly. One can find that when the three design parameters are taken as optimization variables, the minimum total EGR and the maximum SO_2_ yield of the reference reactor restrict each other, i.e., the two different performance objectives cannot achieve the corresponding extremum values at the same time. Then, the second-generation non-dominated solution sequencing genetic algorithm (NSGA-II) is further used to pursue the minimum total EGR and the maximum SO_2_ yield of the reference reactor by taking the three parameters as optimization design variables. After the multi-objective optimization, the reference reactor can be Pareto improved, and the total EGR can be reduced by 9% and the SO_2_ yield can be increased by 14% compared to those of the reference reactor. The obtained results could provide certain theoretical guidance for the optimal design of actual sulfuric acid decomposition reactors.

## 1. Introduction

**Figure 1:**
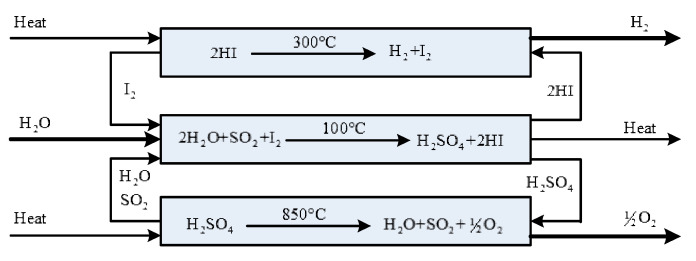
The schematic diagram of S-I thermochemical cycle.

At present, the Hybrid-Sulphur (H-S) thermochemical cycle and the Sulphur-Iodine (S-I) thermochemical cycle are considered to be the two most promising recycling methods in the preparation of hydrogen from water by thermochemical cycles [1], and the schematic diagram of S-I thermochemical cycle is shown in Figure 1. Both the H-S and the S-I cycles contain the sulfuric acid decomposition process. Therefore, it is important and necessary to improve the performance of the sulfuric acid decomposition process.

The S-I thermochemical cycle consists of three main chemical reactions: (1) the endothermic decomposition of hydrogen iodide in gas phase; (2) the spontaneous absorption of sulfur dioxide in liquid phase; (3) the sulfuric acid decomposition reaction. The corresponding reaction equations are given as follows:

(I) $$H_{2}{\mathrm{SO}}_{4}\overset{800K}{\to }{\mathrm{SO}}_{3}+H_{2}O$$

(II) SO3→1100KSO2+12O2

Reaction type (I) is the spontaneous decomposition of H_2_SO_4_ into SO_3_ and H_2_O at 400–500 °C. Reaction type (II) is the reaction of SO_3_ over 750 °C to produce SO_2_ and O2 under the action of a catalyst. In this process, a great deal of heat is consumed, which is also the main energy consumption process in the S-I and H-S thermochemical cycles.

In the aspect of thermodynamic analysis and optimization of sulfuric acid decomposition, Van der ham et al. [1] assumed that the reaction mixture satisfies the ideal gas equation of state, established the physical model of sulfuric acid decomposition reaction, and analyzed the minimization of entropy generation rate (EGR) of a sulfuric acid decomposition reactor by using the optimal control theory. Kuchi et al. [2] carried out a numerical simulation of a high-temperature shell and tube heat exchanger and decomposer, investigated the fluid flow, heat transfer, and chemical reaction processes in the decomposer by using the porous media method, and established a two-dimensional axisymmetric tubular plug-flow reactor model. Ponyavin et al. [3] studied the sulfuric acid decomposer process in a high-temperature ceramic heat exchanger and established a three-dimensional calculation model of the reactor. Van der ham et al. [4] further compared two methods to improve the efficiency of sulfuric acid decomposition reactor and proposed two design schemes to improve the efficiency of the reactor. On the basis of Ref. [1], Wang et al. [5,6] optimized the decomposition of sulfuric acid in the tubular plug-flow reactor with the goal of maximum yield [5], further analyzed the influences of the design parameters of the reactor on the SO_2_ yield and specific EGRs [6], and obtained the optimal parameters corresponding to the minimum specific EGRs.

Many scholars have optimized other types of thermochemical reaction processes by using the theory and method of finite-time thermodynamics (FTT) [7,8,9,10,11,12,13,14,15,16,17,18,19,20,21,22]. For example, Wang et al. [23] investigated the isotherm chemical reaction *A*⇔*B*⇔*C* and obtained the best concentration configuration of the reaction. Johannessen and Kjelstrup [24] studied the EGR minimization of sulfur dioxide oxidation process. The second-generation non-dominated solution sequencing genetic algorithm (NSGA-II) has been widely used in multi-objective optimization of various engineering problems [25,26,27,28,29,30].

On the basis of Refs. [1,5,6], this paper will further analyze the effects of reactant inlet temperature, pressure, and total molar flow rate on total EGR and SO_2_ yield, and perform the multi-objective optimization of the process by using the NSGA-II algorithm by applying FTT.

## 2. Modeling of the Sulfuric Acid Decomposition Process

A reference reactor used in the performance analysis and optimization as well as the kinetics and thermodynamics models will be introduced in this section.

### 2.1. Reference Reactor

**Figure 2:**
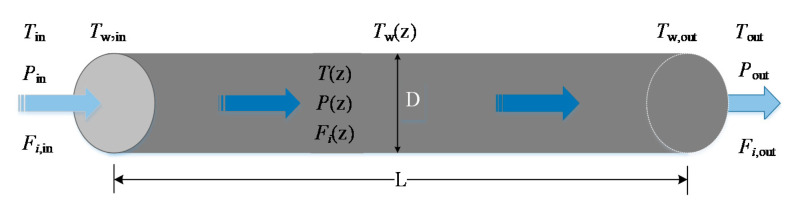
Schematic of tubular plug-flow reactor.

The model of a tubular plug-flow reactor for sulfuric acid decomposition is shown in Figure 2. It is assumed that the temperature (*T*_w_) of the outer wall of tubular plug-flow reactor does not change with time and its distribution is linear along the axial direction of the reactor. The distribution follows $T_{w}=975+148z/L$ (K). The reaction mixture in the reactor is regarded as an ideal gas and only flows along the axial direction of the reactor. The radial concentration gradient and temperature gradient of the reaction mixture in the reactor are ignored without both radial diffusion and back-mixing. The total molar flow rate and velocity of the reaction mixture at the cross-section of the reactor are as follows:

(1) $$F_{tot}=\sum_{i}F_{i}$$

(2) $$v=\frac{F_{tot}}{A_{c}}\frac{R\;T}{P\times 10^{5}}$$

where $F_{i}$ is the molar flow rate of reaction component i, i.e., H_2_SO_4_, SO_3_, H_2_O, SO_2_ and O_2_; $A_{c}$ is the radial cross section area of the reactor, and R is the universal gas constant.

The data of catalyst selection, reactor structure, and thermodynamic parameters of the reaction mixture are determined according to Ref. [1], as listed in Table 1.

### 2.2. Models of Kinetics and Thermodynamics

The fluid flow, heat transfer, and chemical reaction of the reaction mixture in a tubular plug-flow reactor follow momentum, energy, and mass conservation equations, respectively, which are given by:

(3) $$\frac{dP}{dz}=-\left[\frac{150\eta }{D_{p}^{2}}\frac{{\left(1-\varepsilon \right)}^{2}}{\varepsilon^{3}}+\frac{1.75\rho_{\mathrm{in}}v_{\mathrm{in}}}{D_{p}}\frac{1-\varepsilon }{\varepsilon^{3}}\right]v$$

(4) $$\frac{dT}{dz}=\frac{\pi DJ_{q}+A_{c}\rho_{p}\sum_{j}\left[r_{m,j}(-\Delta_{r}H_{j})\right]}{\sum_{i}\left(F_{i}C_{p,i}\right)}$$

(5) $$\frac{dF_{H_{2}{\mathrm{SO}}_{4}}}{dz}=-A_{c}\rho_{p}r_{m,1}$$

(6) $$\frac{dF_{H_{2}O}}{dz}=A_{c}\rho_{p}r_{m,1}$$

(7) $$\frac{dF_{{\mathrm{SO}}_{3}}}{dz}=A_{c}\rho_{p}\left(r_{m,1}-r_{m,2}\right)$$

(8) $$\frac{dF_{{\mathrm{SO}}_{2}}}{dz}=A_{c}\rho_{p}r_{m,2}$$

(9) $$\frac{dF_{O_{2}}}{dz}=\frac{1}{2}A_{c}\rho_{p}r_{m,2}$$

where $\rho_{\mathrm{in}}$ and $v_{\mathrm{in}}$ are the density and flow velocity of the reaction mixture on the entrance section, respectively; subscript j = 1, 2 represents the reaction types (I) and (II); $r_{m,j}$ is the reaction rate of mass per unit catalyst, and they are $r_{m,1}=r_{1}/\rho_{p}$ and $r_{m,2}=r_{2}$; $C_{p,i}$ and $\Delta_{r}H_{j}$ are the component molar constant-pressure heat capacity and the reaction enthalpy of the reaction type j, and their expressions are given in the Appendix A.

The heat transfer from the heat source outside the tube to the reaction mixture inside the tube follows Newtonian heat transfer law:

(10) $$J_{q}=U\left(T_{w}-T\right)$$

For different reaction conditions and mechanisms, the driving force in the kinetic equation could be written as different mathematical forms, and the corresponding coefficients in the kinetic equation should be determined by experiments and also be different for different choices of the driving force. According to Ref. [1], the condition that the chemical reaction occurred at the vicinity of the equilibrium is assumed to be satisfied, and all components are assumed to have stoichiometric reaction order, so the chemical reaction rates of reaction types (I) and (II) are as follows:

(11) $$r_{1}=k_{1}\left(P_{H_{2}{\mathrm{SO}}_{4}}-\frac{P_{H_{2}O}P_{{\mathrm{SO}}_{3}}}{K_{1}}\right)$$

(12) $$r_{2}=k_{2}\left(P_{{\mathrm{SO}}_{3}}-\frac{P_{{\mathrm{SO}}_{2}}\sqrt{P_{O_{2}}}}{K_{2}}\right)$$

where $k_{1}$ and $k_{2}$ are the reaction rate constants of reaction types (I) and (II), according to Ref. [1], $k_{1}=10^{-3}\mathrm{mol}(SO_{3})/(\mathrm{Pa}\cdot m^{3}\cdot s)$, $k_{2}=4.7\times 10^{-3}\exp^{\left(\frac{-99\cdot 10^{3}}{RT}\right)}\mathrm{mol}(SO_{3})/(\mathrm{Pa}\cdot \mathrm{kg}\cdot s)$; *P* represents the partial pressure of the corresponding component; $K_{j}=\exp\left(\frac{\Delta_{r}G_{T,j}^{{}^{\circ}}}{-RT}\right)$ is the equilibrium constant of the chemical reaction type j; $\Delta_{r}G_{T,j}^{{}^{\circ}}$ is the standard Gibbs free enthalpy of the reaction type j, and the expression is given in the Appendix A. The driving force in the kinetic Equation (12) is written as $r_{2}=k_{2}\left(P_{{\mathrm{SO}}_{3}}-P_{{\mathrm{SO}}_{2}}\sqrt{P_{O_{2}}}/K_{2}\right)$, and effects of the different forms of the driving force on the optimization results will be considered in another paper in the future.

The SO_2_ yield of the tubular plug-flow reactor is as follows:

(13) $$\Delta F_{{\mathrm{SO}}_{2}}=F_{{\mathrm{SO}}_{2},\mathrm{out}}-F_{{\mathrm{SO}}_{2},\mathrm{in}}$$

The local EGR of the tubular plug-flow reactor is as follows:

(14) $$\begin{array}{cc} \sigma_{\mathrm{tot}} & =\sigma_{\mathrm{ht}}+\sigma_{f}+\sigma_{\mathrm{cr}}\\ & =\pi DJ_{q}\left(\frac{1}{T}-\frac{1}{T_{w}}\right)+A_{c}v\left[-\frac{1}{T}\left(\frac{dP}{dz}\right)\right]+A_{c}\rho_{b}\sum_{j}r_{m,j}\left(-\frac{\Delta_{r}G_{j}}{T}\right) \end{array}$$

where subscripts ht, f, and cr represent the local EGRs of heat transfer, fluid flow, and chemical reaction, respectively.

The total EGR is obtained by integrating the local EGR, i.e.,

(15) $$\Sigma_{tot}=\int_{0}^{L}\sigma_{tot}dz$$

## 3. Parameter Analyses of Sulfuric Acid Decomposition Reactor

By changing the inlet parameters of the reference reactor, including the inlet temperature *T*_in_, pressure *P*_in_ and the total molar flow rate *F*_tot,in_, the total EGR and the SO_2_ yield of the reference reactor are analyzed, and the influences of the initial inlet conditions on the two performance objectives can be obtained. The variation ranges of the initial inlet parameters are: 750 K ≤ *T*_in_ ≤ 900 K, 4 MPa ≤ *P*_in_ ≤ 9.5 MPa, and 0.0027 mol/s ≤ *F*_tot,in_ ≤ 0.1 mol/s.

**Figure 3:**
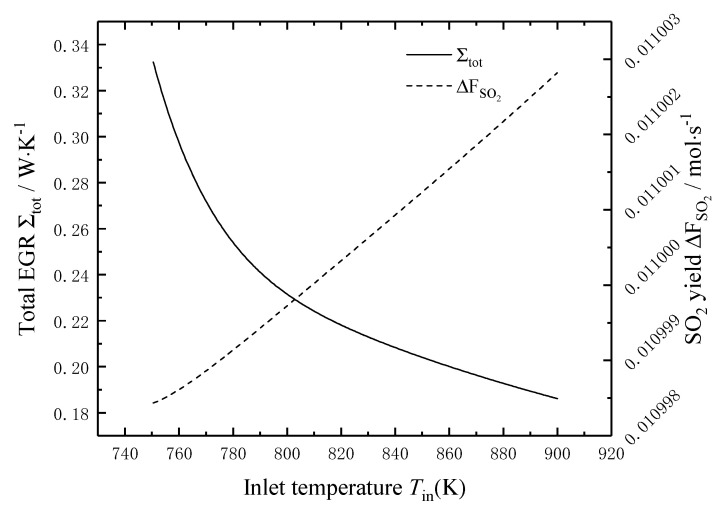
The effects of $T_{\mathrm{in}}$ on the total EGR and the SO_2_ yield.

Figure 3 shows the effects of the temperature *T*_in_ of the reaction mixture on the total EGR and the SO_2_ yield. It can be seen that the total EGR decreases nonlinearly with the increase of the temperature *T*_in_, and the decreasing trend is fast firstly and then slow; when the temperature *T*_in_ increases from 750 °C to 900 °C, the total EGR decreases from 0.331 W/K to 0.189 W/K, i.e., decreases by 43%. The main reason is that with the temperature *T*_in_ of the reaction mixture increases, the heat transfer temperature difference between the reaction mixture and the external heat source decreases, which reduces the local EGR of heat transfer and the total EGR. The SO_2_ yield increases very slowly with the increase of the temperature *T*_in_, and when the temperature *T*_in_ increases from 750 °C to 900 °C, the SO_2_ yield increases by only 0.4%. It can be seen that the total EGR can be reduced by increasing the temperature *T*_in_ of the reaction mixture, i.e., the irreversibility of the sulfuric acid decomposition process could be reduced by increasing the *T*_in_ of the reaction mixture. However, it is not significant to increase the SO_2_ yield by increasing the temperature *T*_in_ of the reaction mixture.

**Figure 4:**
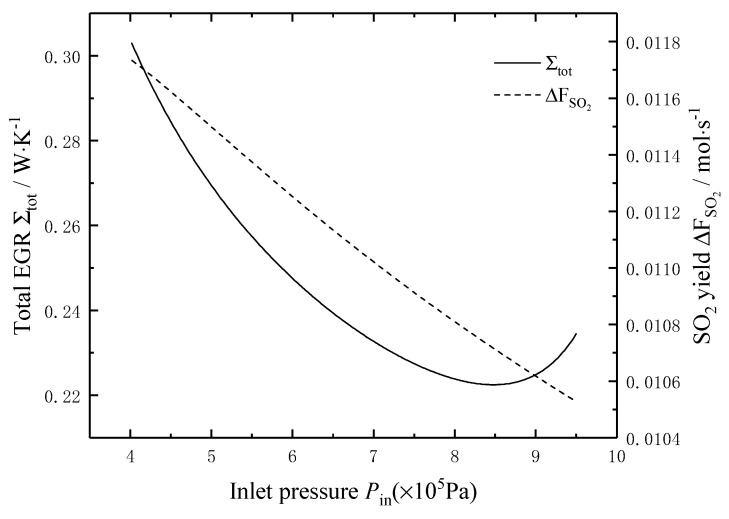
Effects of $P_{\mathrm{in}}$ on the total EGR and the SO_2_ yield.

Figure 4 shows the effects of the pressure *P*_in_ of the reaction mixture on the total EGR and the SO_2_ yield. It can be seen that the curve of the total EGR is concave and parabolic-like with the increase of the pressure *P*_in_, and the minimum value is 0.224 W/K when the pressure *P*_in_ is about 0.85 MPa. The SO_2_ yield decreases linearly with the increase of the pressure *P*_in_. When the pressure *P*_in_ increases from 0.4 MPa to 1 MPa, the SO_2_ yield decreases from 0.0118 mol/s to 0.0105 mol/s, i.e., decreases by 11.02%.

**Figure 5:**
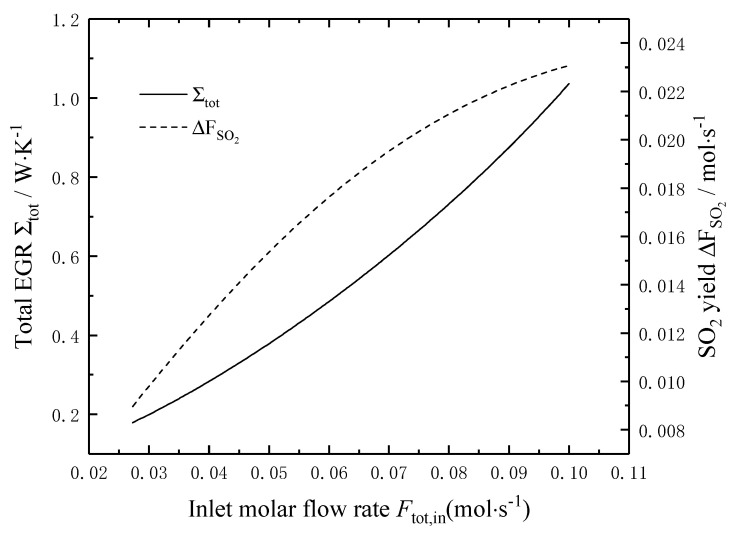
Effects of $F_{\mathrm{tot},\mathrm{in}}$ on the total EGR and the SO_2_ yield.

Figure 5 shows the effects of the molar flow rate *F*_tot,in_ of the reaction mixture on the total EGR and the SO_2_ yield. It can be seen that the total EGR and the SO_2_ yield increase with the increase of the molar flow rate *F*_tot,in_, and the minimum total EGR and the maximum SO_2_ yield are mutually restricted. When the molar flow rate *F*_tot,in_ increases from 0.027 mol/s to 0.10 mol/s, the total EGR and the SO_2_ yield increases by 4.8 times and 1.8 times, respectively.

## 4. Multi-Objective Optimization and Result Analyses

From the analyses in Section 3, when the three inlet parameters are chosen as optimization variables, and the minimum total EGR and the maximum SO_2_ yield are taken as optimization objectives, respectively, there is no optimal solution to achieve the extremum values of the total EGR and SO_2_ yield at the same time. Therefore, how to select the appropriate initial inlet conditions to achieve the relative optimal total EGR and SO_2_ yield is very important. The NSGA-II algorithm is one of the excellent algorithms to solve multi-objective optimization problems, and can give a series of non-inferior solutions (solutions that cannot be optimized for arbitrary objectives without making other objectives worse) of multi-objective problems. The corresponding improvement process is called Pareto improvement, the corresponding set of non-inferior solutions is called the Pareto-optimal solution set, and the corresponding objective function solution is called the Pareto-optimal front.

**Figure 6:**
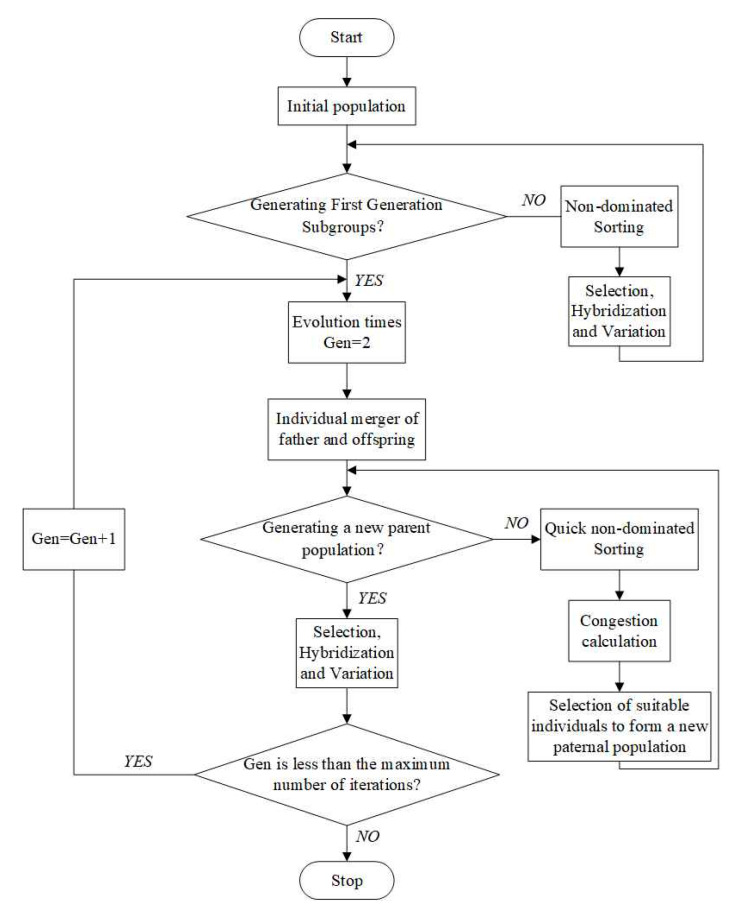
Basic flow chart of NSGA-II algorithm.

Figure 6 shows the flow chart of the NSGA-II algorithm. In this section, all of the *T*_in_, *P*_in_ and *F*_tot,in_ are taken as the optimization variables to minimize the total EGR and maximize the SO_2_ yield. The optimization intervals of the variables are consistent with the previous single-variable analysis.

**Figure 7:**
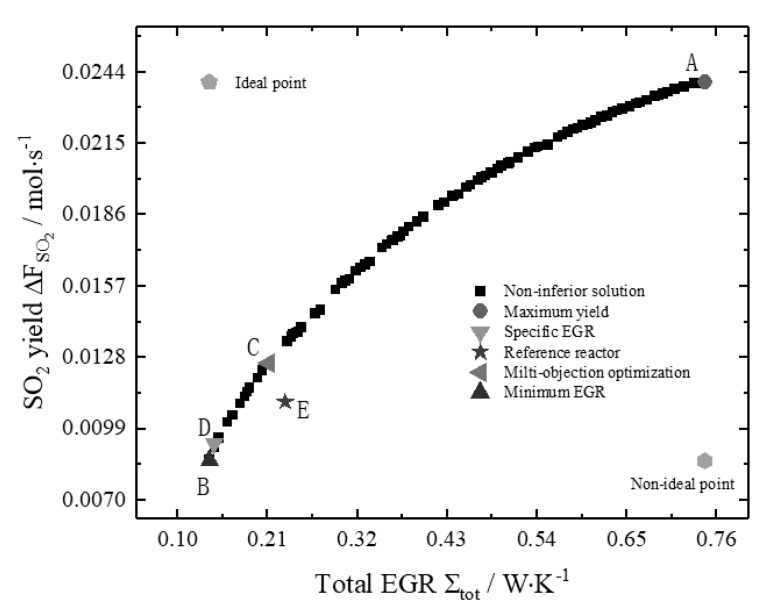
Pareto optimal frontiers of reference reactor.

Figure 7 is Pareto optimal frontier of a reference reactor based on the objective of minimizing total EGR and maximizing SO_2_ yield, where points A and B represent the solution of the maximum SO2 yield and the minimum total EGR, respectively. At point A, the weighting coefficient of SO_2_ yield in multi-objective optimization is 1, and the weighting coefficient of total EGR is 0, it is also the solution of maximizing the SO_2_ yield. Similarly, point B is the solution of minimizing the total EGR. From Figure 7, it can be seen that the minimum total EGR and the maximum SO_2_ yield are mutually constrained, and they cannot achieve the extremum values at the same time. Only the relative optimal solutions of the two objectives under different weighting coefficients can be found, that is, the non-inferior solution. One can select the appropriate optimal solution from the Pareto-optimal solution set according to different needs to meet the different demands of decision-making purposes. Commonly used multi-objective decision-making methods are Shannon, LINMAP, and TOPSIS, but in the actual decision-making process, decision-making is usually based on actual engineering experience and personal preferences of decision-makers, there is no universal way to make decisions.

In this paper, in order to facilitate the comparison with the reference reactor, a suitable multi-objective decision point (point C) is selected for comparison. Because the solution of the minimum specific EGRs is the solution of the total EGR and the yield under a certain ratio, the decision point of the minimum specific EGR must be on the Pareto-optimal front, which can be used as an important basis to verify the accuracy of the NSGA-II algorithm results.

**Figure 8:**
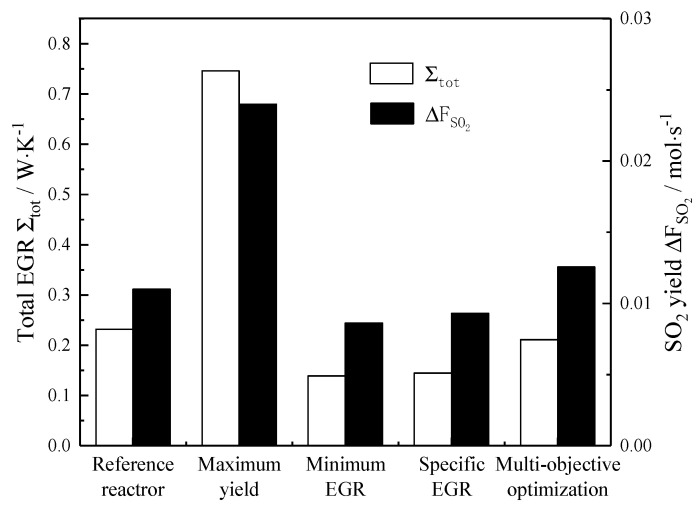
Comparison of total EGR and the yield of optimized objectives.

Figure 8 is the bar chart of the target value of the reference reactor under optimization and non-optimization. Table 2 lists the results of each optimization target condition. It can be seen that compared with the reference reactor, the SO_2_ yield of the reactor with the maximum yield increases by 118%, but the total EGR increases by 222%; the total EGR of the minimum EGR reactor decreases by 40%, and the corresponding SO_2_ yield also decreased by 22%; the total EGR and the SO_2_ yield of the reactor with the minimum specific EGR decrease by 38% and 16%, respectively. From Figure 7, it can be easily concluded that the reference reactor is not located at the Pareto optimal frontier, so the reference reactor can be optimized by Pareto improvement. A non-inferior solution (point C) is obtained by the multi-objective optimization method, in which the total EGR of the reactor decreases by 9% and the SO_2_ yield of the reactor increases by 14% compared to the reference reactor. Also, from Figure 7, it can be seen that a series of non-inferior solutions located at the upper left of the decision point (point E) of the reactor have good properties of reducing the total EGR and increasing the SO_2_ yield.

**Figure 9:**
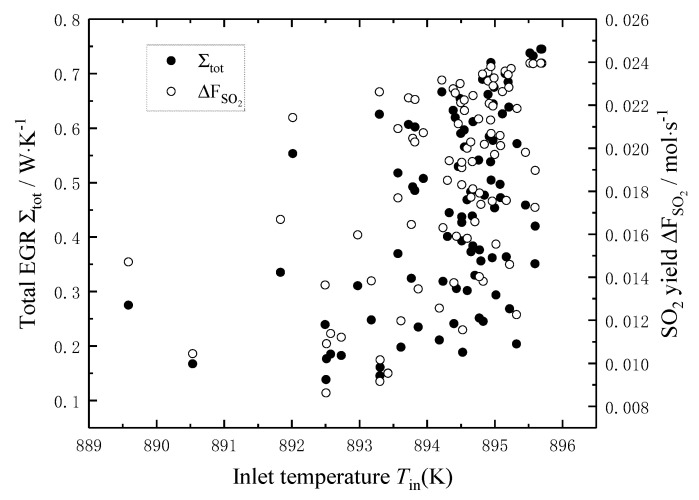
Distribution of inlet temperature in Pareto-optimal fronts.

**Figure 10:**
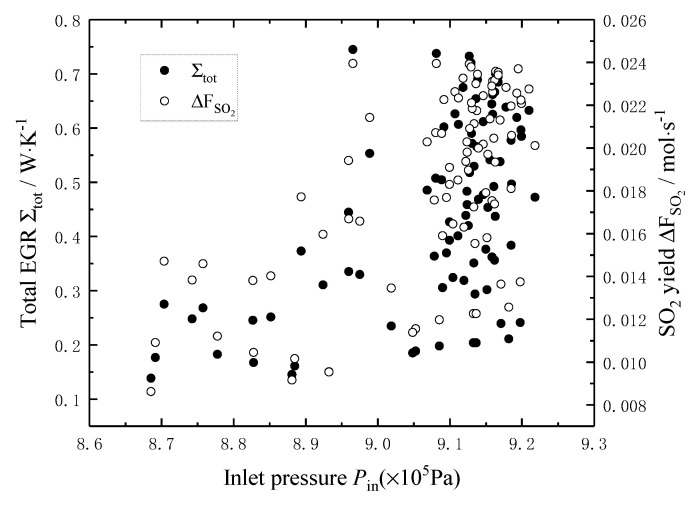
Distribution of inlet pressure in Pareto-optimal fronts.

**Figure 11:**
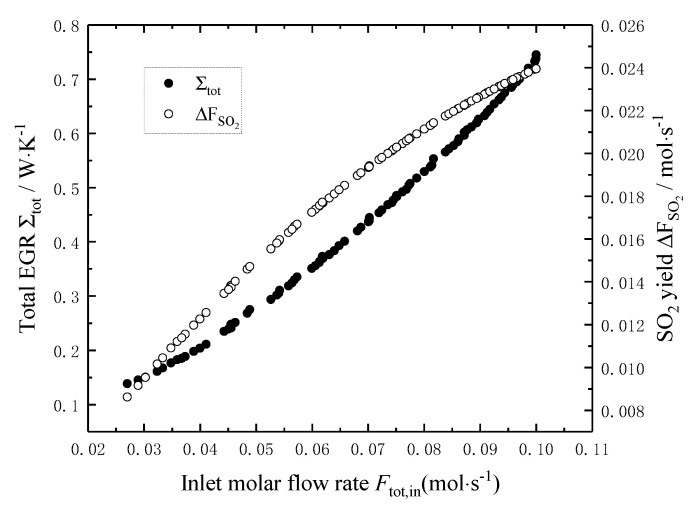
Distribution of total inlet molar flow rate in Pareto-optimal fronts.

Figure 9, Figure 10 and Figure 11 show the distribution of the *T*_in_, *P*_in_ and *F*_tot,in_ in Pareto-optimal fronts, and the black and white dots in the figures represent the total EGR and the SO_2_ yield, respectively, which exist in pairs. As seen from Figure 9 and Figure 10, the *T*_in_ and *P*_in_ of the reaction mixture in Pareto-optimal fronts are mainly distributed in high-temperature (892–896 K) and high-pressure (9.0–9.2 bar) area, so increasing the *T*_in_ and *P*_in_ of the reaction mixture is an important means for Pareto improvement. Figure 11 shows that the *F*_tot,in_ of the reaction mixture in Pareto-optimal fronts distributes uniformly in its optimal range, which indicates that adjusting the *F*_tot,in_ of the reaction mixture in Pareto-optimal fronts is an important means to reconcile the contradiction between the minimum total EGR and the maximum SO_2_ yield.

## 5. Conclusions

In this paper, the effects of reaction mixture inlet parameters on the total EGR and SO_2_ yield of the tubular plug-flow sulfuric acid decomposition reactor are analyzed, and the multi-objective optimization for the two performance objectives are carried out by using FTT. The results show that:

(1) When the *T*_in_ increases from 750 °C to 900 °C, the total EGR decreases by 43% and the SO_2_ yield increases by 0.4%. When the *P*_in_ increases from 0.4 MPa to 1 MPa, the curve of the total EGR versus the *P*_in_ is a concave parabolic-like, the minimum value of the total EGR is 0.224 W/K when the *P*_in_ equals to 0.85 MPa, and the corresponding SO_2_ yield decreases by 11%. When the *F*_tot,in_ increases from 0.027mol/s to 0.10mol/s, the total EGR and the SO_2_ yield increase by 4.8 times and 1.8 times, respectively.
(2) The reference reactor can be Pareto improvement, one of the non-inferior solutions can reduce the total EGR by 9% and increase the SO_2_ yield by 14% compared to those of the reference reactor.
(3) FTT is a powerful theoretical tool for the performance analysis and optimization of tubular plug-flow sulfuric acid decomposition reactor. The NSGA-II algorithm is an effective mathematical tool for the multi-objective optimization of tubular plug-flow sulfuric acid decomposition reactor. The Pareto-optimal fronts obtained in this paper has a certain theoretical guiding significance for the optimal designs of the actual sulfuric acid decomposition reactors.

## Figures and Tables

**Table 1:** Parameters of the reference reactor.

Parameter	Symbol	Value
Overall heat transfer coefficient/$J/\left(K\cdot m^{2}\cdot s\right)$	U	170
Dynamic viscosity/kg/(m·s)	η	4 × 10^−5^
Catalyst bed porosity	ε	0.45
Catalyst pellet density$/\mathrm{kg}/m^{3}$	$\rho_{p}$	4200
Catalyst pellet diameter/m	$D_{p}$	0.003
Inner diameter of reactor/m	D	0.030
Length of reactor/m	L	3.090
Inlet temperature/K	*T*_in_	800
Inlet pressure/bar	*P*_in_	7.1
Inlet total molar flow rate	*F*_tot,in_	0.034
Molar fraction of inlet H_2_SO_4_	$F_{H_{2}{\mathrm{SO}}_{4},\mathrm{in}}$	0.094
Molar fraction of inlet SO_3_	$F_{{\mathrm{SO}}_{3},\mathrm{in}}$	0.425
Molar fraction of inlet H_2_O	$F_{H_{2}O,\mathrm{in}}$	0.481
Molar fraction of inlet SO_2_	$F_{{\mathrm{SO}}_{2},\mathrm{in}}$	0.000
Molar fraction of inlet O_2_	$F_{O_{2},\mathrm{in}}$	0.000

**Table 2:** Calculation results of each target.

	Reactor Inlet Parameters			EGR		SO_2_ Yield	
	Temperature *T*_in_(K)	Pressure $P_{\mathrm{in}}(1\times 10^{5}\mathrm{Pa})$	Molar Rate $F_{tot,in}$$\left(\mathrm{mol}\cdot s^{-1}\right)$	$\Sigma_{tot}/W\cdot K^{-1}$		$\Delta F_{SO_{2}}/mol\cdot s^{-1}$	
Reference reactor	800	7.10	0.034	0.2316	——	0.01100	——
Maximum yield	896	8.97	0.010	0.7450	↑222%	0.02395	↑118%
Minimum EGR	893	8.69	0.027	0.1388	↓40%	0.00862	↓22%
Specific EGR	900	8.62	0.030	0.1446	↓38%	0.00930	↓16%
Multi-objective optimization	894	9.18	0.041	0.2111	↓9%	0.01256	↑14%

## Nomenclature

A	area $m^{2}$
$C_{P}$	molar constant-pressure heat capacity, KJ/(mol·K)
$D_{p}$	catalyst pellet diameter, m
*F*	molar flow rate, mol/s
$J_{q}$	heat flux density, W/m^2^
*K*	equilibrium constant
L	length, m
P	pressure, bar
R	universal gas constant, J/(mol·K)
*r*	reaction rate, mol/(kg·s)
T	temperature, K
v	flow velocity, m/s
z	length, m
**Greek letters**	
ε	catalyst bed porosity
η	dynamic viscosity, kg/(m·s)
κ	rate constant of chemical reaction
$\nu_{i}$	the stoichiometric number of reaction component i
ρ	density, $\mathrm{kg}\cdot m^{-3}$
σ	local EGR, J/K
Σ	total
$\Delta_{r}G$	Gibbs free energy change of chemical reaction, J
$\Delta_{r}H$	enthalpy change of chemical reaction, J
**Subscripts**	
c	cross section of tubular plug-flow reactor
cr	chemical reaction
f	fluid flow
ht	heat transfer
i	component
in	inlet
j	reaction types (I) and (II)
out	outlet
p	catalyst pellet
q	quantity of heat
r	reaction
tot	total
w	wall of tubular plug-flow reactor
**Abbreviations**	
EGR	entropy generation rate
FTT	finite-time thermodynamics
H-S	hybrid-Sulphur thermochemical cycle
NSGA-II	second generation non-dominated solution sequencing genetic algorithm
S-I	sulphur-Iodine thermochemical cycle

## Appendix A

According to the Refs. [31], the component molar constant-pressure heat capacity, molar enthalpy and molar Gibbs energy can be calculated by the following formula:

(A1) $$C_{p,i}^{{}^{\circ}}=A_{i}+B_{i}\frac{T}{1000}+C_{i}{\left(\frac{T}{1000}\right)}^{2}+D_{i}{\left(\frac{T}{1000}\right)}^{3}+E_{i}{\left(\frac{1000}{T}\right)}^{2}$$

(A2) $$H_{T,i}^{{}^{\circ}}=A_{i}\frac{T}{1000}+\frac{B_{i}}{2}{\left(\frac{T}{1000}\right)}^{2}+\frac{C_{i}}{3}{\left(\frac{T}{1000}\right)}^{3}+\frac{D_{i}}{4}{\left(\frac{T}{1000}\right)}^{4}-E_{i}\left(\frac{1000}{T}\right)+F_{i}$$

(A3) $$S_{T,i}^{{}^{\circ}}=A_{i}In\left(\frac{T}{1000}\right)+B_{i}\frac{T}{1000}+\frac{C_{i}}{2}{\left(\frac{T}{1000}\right)}^{2}+\frac{D_{i}}{3}{\left(\frac{T}{1000}\right)}^{3}-\frac{E_{i}}{2}{\left(\frac{1000}{T}\right)}^{2}+G_{i}$$

(A4) $$\Delta_{r}H_{T}^{{}^{\circ}}=\sum_{i}v_{i}H_{T,i}^{{}^{\circ}}$$

(A5) $$\Delta_{r}S_{T}^{{}^{\circ}}=\sum_{i}v_{i}S_{T,i}^{{}^{\circ}}$$

(A6) $$\Delta_{r}G_{T}^{{}^{\circ}}=\Delta_{r}H_{T}^{{}^{\circ}}-T\Delta_{r}S_{T}^{{}^{\circ}}$$

where $A_{i}$∼$G_{i}$ are the thermodynamic coefficients of the formula, which are listed in Table A1; $v_{i}$ is the stoichiometric number of reaction component i.

**Table A1:** Thermodynamic coefficients.

Gas	$M_{W,i}/kg\cdot mol^{-1}$	$A_{i}$	$B_{i}$	$C_{i}$	$D_{i}$	$E_{i}$	$F_{i}$	$G_{i}$	$T_{\min}/K$	$T_{\max}/K$
**SO_2_**	$6.40\times 10^{-2}$	21.430	74.351	−57.752	16.355	0.087	−305.769	254.887	298	1200
**O_2_**	$3.20\times 10^{-2}$	29.659	6.137	−1.187	0.096	−0.220	−9.861	237.948	298	6000
**SO_3_**	$8.01\times 10^{-2}$	24.025	119.461	−94.387	26.926	−0.118	−407.853	253.51	298	1200
**H_2_O**	$1.80\times 10^{-2}$	30.092	6.833	6.793	−2.534	0.082	−250.881	223.397	500	1700
**H_2_SO_4_**	$9.81\times 10^{-2}$	47.289	190.331	−148.123	43.868	−0.740	−758.953	301.296	298	1200
